# Impact of surface coating on electrochemical and thermal behaviors of a Li-rich Li_1.2_Ni_0.16_Mn_0.56_Co_0.08_O_2_ cathode†

**DOI:** 10.1039/d0ra02060e

**Published:** 2020-04-17

**Authors:** Umair Nisar, Ramesh Petla, Sara Ahmad Jassim Al-Hail, Aisha Abdul Quddus, Haya Monawwar, Abdul Shakoor, Rachid Essehli, Ruhul Amin

**Affiliations:** Center for Advanced Materials (CAM), Qatar University Doha Qatar shakoor@qu.edu.qa; Qatar Environment and Energy Research Institute (QEERI), Hamad Bin Khalifa University, Qatar Foundation Doha 34110 Qatar; Department of Chemical Engineering, College of Engineering, Qatar University Doha Qatar; Department of Electrical Engineering, College of Engineering, Qatar University Doha Qatar; Energy and Transportation Science Division, Oak Ridge National Laboratory Oak Ridge TN USA aminr@ornl.gov

## Abstract

Lithium-rich layered oxide materials are considered as potential cathode materials for future high-performance lithium-ion batteries (LIBs) owing to their high operating voltage and relatively high specific capacity. However, perceptible issues such as poor rate performance, poor capacity retention, and voltage degradation during cycling need to be improved before the successful commercialization of the material. In this report, zirconia coated Li_1.2_Ni_0.16_Mn_0.56_Co_0.0_8O_2_ (NMC) (where ZrO_2_ = 1.0, 1.5 and 2.0 wt%) materials are synthesized using a sol–gel assisted ball milling approach. A comparison of structural, morphological and electrochemical properties is examined to elucidate the promising role of ZrO_2_ coating on the performance of the NMC cathode. A uniform and homogeneous ZrO_2_ coating is observed on the surface of NMC particles as evident by TEM elemental mapping images. The ZrO_2_ coated NMCs exhibit significantly improved electrochemical performance at a higher C-rate as compared to pristine material. 1.5% ZrO_2_ coated NMC demonstrates better cycling stability (95% capacity retention) than pristine NMC (77% capacity retention) after 50 cycles. All ZrO_2_ coated NMC materials demonstrated improved thermal stability compared to pristine material. The difference in onset temperature of 2 wt% ZrO_2_ coated and pristine NMC is 20 °C. The improved electrochemical performance of ZrO_2_ coated NMC can be attributed to the stabilization of its surface structure due to the presence of ZrO_2_.

## Abstract 2

ZrO_2_ coated Li_1.2_Ni_0.16_Mn_0.56_Co_0.08_O_2_ (NMC) materials with improved electrochemcial performance.

## Graphical abstract

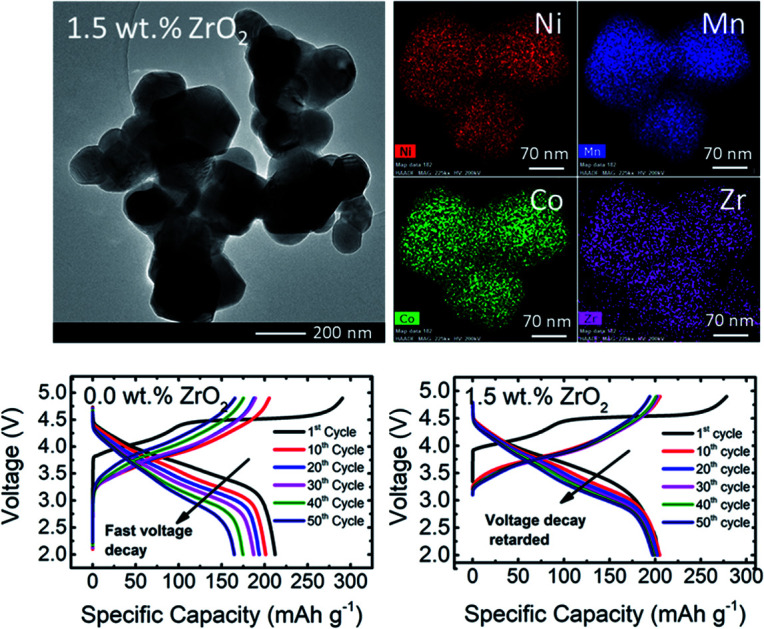

## Introduction

Due to continuous emissions of greenhouse gases and environmental concerns, the world has recognized the alarming consequences of global warming. Therefore, focus has been diverted to explore renewable energy resources which can fulfil future energy demands.^1,2^ In this regard, many efforts have been devoted to utilizing solar, wind or tidal energy as a clean energy source for future needs.^3^ Unfortunately, energy from these sources is not continuous and fluctuates depending on different factors; therefore, the energy from these sources should be preserved in some sort of energy storage system (ESS) and should be readily available when needed.^1,2,4^ Batteries and supercapacitors are well-known energy storage systems and were commercialized long ago.^2,5^ Amongst them, batteries are obviously better energy storage systems owing to their very high energy density as compared to supercapacitors.^2,3,5,6^ Amongst various battery technologies, lithium-ion batteries (LIBs) are at the top of the list due to their higher capacity, high operating voltage, good calendar life, and high energy and power density.^6–12^ However, the currently available LIB technology has several shortfalls such as poor rate performance, and insufficient energy and power density to power future electric vehicles. Moreover, challenges of safety and cost also need to be smartly addressed to fulfil the future energy storage needs.^2,9,11,13^ Nonetheless, a lot of research work and efforts are required to optimize the currently available battery materials and to develop new battery materials that can operate at high voltage, deliver high energy, power density and demonstrate safety and cost-effectiveness.^6,9,14,15^ In the quest to establish high-performance LIBs, several cathode materials have been developed and commercialized in the last couple of decades including LiMn_2_O_4_, LiCoO_2_, LiNi_1/3_Mn_1/3_Co_1/3_O_2_, LiNi_0.5_Mn_1.5_O_4_, LiFePO_4_, LiNi_0.8_Co_0.15_Al_0.05_O_2_ and LiNi_0.5_Co_0.2_Mn_0.3_O_2_.^12,16–19^ All of these materials have already reached their practical energy density limits. However, new materials need to be developed with higher specific capacity (>200 mA h g^−1^), efficiently powering electric cars by battery systems, capable of higher voltage operation (>3.5 V *vs.* Li) and should be cost effective.^10,14,15^ In this regard, lithium-rich layered oxide *x*Li_2_MnO_3_·(1 − *x*)LiMO_2_ (M = Mn, Ni, Co, Fe, *etc.*) materials have been shown huge potential to be considered as high energy density materials for next-generation batteries due to their high operating voltage (>3.6 V *vs.* Li) and very high specific capacities (>250 mA h g^−1^).^7,20–22^ Nonetheless, these materials also have huge scientific challenges that need to be elegantly addressed before their successful commercialization. One of the serious issues of this class of materials is the rapid voltage and capacity degradation with successive cycling.^20–24^ Apart from this, these materials show poor initial coulombic efficiency, poor rate capability, structural instability, and safety issues which retard their commercialization.^20–22^ Most of these problems are linked to the unstable interface between the cathode and the organic electrolyte, especially at high operating voltage leading to the development of unstable solid electrolyte interface (SEI).^25–27^ To address these issues, several strategies have been widely adopted including surface coatings of active materials, elemental doping on Ni, Mn or Co sites and tailoring the particle morphology.^6–8,12,28–33^ Amongst them, the surface coating of active materials has been widely accepted and employed to improve the performance of these materials. Several coating materials such as Al_2_O_3_, SiO_2_, TiO_2_, ZrO_2_, MgO, ZnO, and AlF_3_ have been investigated in different cathode materials.^6,34–38^ These coatings reduce the unwanted side reactions between electrolyte and electrode surface which helps to improve the electrochemical performance.^6,38^ Unfortunately, a detailed study about mechanisms of how these coatings behave and improve the performance of the materials at different operating conditions is still not fully understood and not readily available. Amongst these coating materials, ZrO_2_ coating has shown remarkable potential and several studies have demonstrated the improvement in electrochemical performance of cathode materials with ZrO_2_ coating.^6,36,39^ To the best of our knowledge, the coating of Li_1.2_Ni_0.16_Mn_0.56_Co_0.08_O_2_ (NMC) with ZrO_2_ has not been reported so far indicating a room for its further exploration. Here, ZrO_2_ coated Li_1.2_Ni_0.16_Mn_0.56_Co_0.08_O_2_ (ZrO_2_ = 1.0, 1.5 & 2.0 wt%) materials were synthesized using sol–gel process and ball milling approach. The salient feature of the coating process employed here is its ease of scalability and cost-effectiveness. After synthesis, the materials were characterized using X-ray diffraction (XRD), scanning electron microscopy (SEM), transmission electron microscope (TEM), X-ray photoelectron spectroscopy (XPS). The electrochemical charge/discharge cycles were performed at different current rates to check the stability pristine and coated electrodes. Finally, electrochemical impedance spectroscopy (EIS) was used to study the interfacial kinetics. The obtained results show the pathway to improve the rate capability, cyclability and safety of the NMC materials.

## Experimental

### Materials synthesis

All the materials were synthesized using the sol–gel synthesis method. Firstly, lithium acetate dihydrate, manganese acetate tetrahydrate, nickel acetate tetrahydrate, and cobalt acetate tetrahydrate (Sigma Aldrich) were dissolved in 100 ml of deionized water in the molar ratio of 1.2 : 0.16 : 0.56 : 0.08 respectively. Later, the citric acid (metal ions : citric acid 1 : 1) was added to the precursor solution as a chelating agent. The precursor solution was kept at 70 °C under continuous stirring until completely dried. The dried precursor mixture was then shifted to the conventional oven at 120 °C for 12 hours to remove the traces of water. The precursor mixture was then ground and homogeneously mixed using agate mortar. Finally, the precursor mixture was calcinated at 900 °C for 12 hours in a muffle furnace to synthesize pristine Li_1.2_Ni_0.16_Mn_0.56_Co_0.08_O_2_ (NMC) cathode material. For ZrO_2_ coated Li_1.2_Ni_0.16_Mn_0.56_Co_0.08_O_2_ (NMC), the precursor mixture was decomposed at 450 °C for 6 hours in the air. Then, ZrO_2_ (where ZrO_2_ = 1.0, 1.5 & 2.0 wt%) was added to decomposed precursor material and ball milled for 24 hours using ZrO_2_ grinding media. Finally, the ball-milled materials were sintered at 900 °C for 12 hours in a muffle furnace to synthesized ZrO_2_ coated Li_1.2_Ni_0.16_Mn_0.56_Co_0.08_O_2_ materials.

### Structural, compositional and thermal stability characterization

Powder X-ray diffraction (XRD) measurements were carried out, to identify the phase and purity of synthesized materials, using a PANanlytical diffractometer (Empyrean) equipped with the Cu-Kα radiation. The materials were scanned between 10° ≤ 2*θ* ≤ 90° angular range with the step size of 0.01°. The materials morphology (size and shape) was investigated using field emission scanning electron microscopy (FE-SEM, Nova) and transmission electron microscope (TEM, Talos F200X, FEI). TEM was further utilized to map the elemental distribution on the surface of the particles. X-ray Photoelectron Spectroscopy (XPS) (Thermo-Scientific-Sigma Probe) was further used to confirm the presence of the ZrO_2_ on the surface of the NMC particles. The thermal stability of pristine and ZrO_2_ coated materials in the charged state was evaluated using differential scanning calorimetry (DSC, 8500, PerkinElmer). The cells were charged at 0.2C to 4.7 V and held there for 30 min. Later, these cells were disassembled in the argon-filled glove box. The charged material was scratched from the aluminum foil, and the excess electrolyte was removed from the surface of the electrode. Later, this material was transferred to high-pressure stainless-steel pan with gold-plated copper seal to perform DSC analysis. The measurements were carried out using a scan rate of 5 °C min^−1^ in the temperature range of 50–350 °C.

### Electrode fabrication

The cathodes for the electrochemical testing were fabricated by making a slurry consisting of active material (pristine NMC and ZrO_2_ coated NMC), conductive carbon (Super-P) and polyvinylidene fluoride (PVDF) (80 : 10 : 10) in 1-methyl-2-pyrrolidone (NMP). The slurry was later cast on aluminum foil using a doctor blade technique. The casted electrodes were dried in an oven at 80 °C overnight to remove 1-methyl-2-pyrrolidone (NMP). Later, the electrodes of 14 mm diameter were punched through these casted electrodes, which were then transferred to a vacuum oven at 120 °C to remove the traces of moisture. The electrodes were then transferred to argon-filled glovebox for cell fabrication.

### Electrochemical measurements

Electrochemical testing was carried out using 2032-type coin cells assembled in argon-filled glovebox using lithium metal foil as a negative electrode (anode). Celgard 2325 was used as a separator, and the synthesized pristine NMC and ZrO_2_ coated NMC were used as the cathodes. The electrolyte was composed of 1 M LiPF_6_ dissolved in a mixture of ethylene carbonate (EC) and dimethyl carbonate (DMC) (1 : 1 by v/v). All galvanostatic charge/discharge measurements were performed at room temperature (25 °C) in the voltage window of 2.0–4.9 V using battery cycler (WonAtech WBCS 3000L). Galvanostatic Intermittent Titration Technique (GITT) measurements were carried out using Solartron battery cycler (1470E) as already reported in detail in our previous publication.^40^ The electrochemical impedance measurements (EIS) were performed between the frequency range of 2–5 mHz using sinusoidal voltage amplitude of 10 mV, and the obtained data was fitted using Z-view software using an equivalent circuit model.

## Results and discussion

**Fig. 1:**
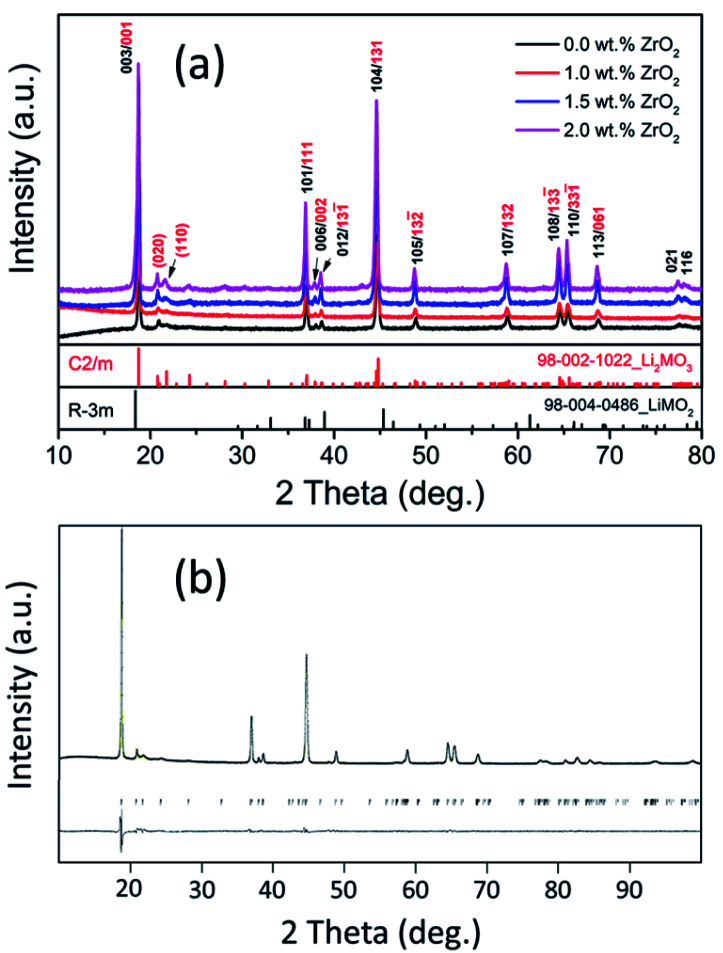
(a) XRD patterns for pristine and ZrO_2_ coated Li_1.2_Ni_0.16_Mn_0.56_Co_0.08_O_2_ (ZrO_2_ = 1.0, 1.5 & 2.0 wt%), (b) Rietveld refinement of Li_1.2_Ni_0.16_Mn_0.56_Co_0.08_O_2_ with observed and calculated spectra.

Fig. 1(a) shows the X-ray diffraction (XRD) patterns for pristine and ZrO_2_ coated NMC (ZrO_2_ = 1.0, 1.5 & 2.0 wt%) materials which confirm the formation of highly crystalline and pure materials without any impurity phases. The ZrO_2_ peaks are not visible in the XRD patterns that may be due to its small amount present in the materials. The Li-rich NMC materials are composed of the mixture of two-phases (Li_2_MO_3_ and LiMO_2_) and can be indexed to *R*3̄*m* and *C*2/*m* space groups, respectively, as shown in Fig. 1(a).^7^Fig. 1(b) shows the Rietveld refinement pattern of pristine NMC. All the calculated and observed peaks match well with each other confirming the high purity of the synthesized materials. The calculated cell parameters for pristine NMC are shown in Table 1, which matches well with the previous reports.^40^

**Table 1:** Cell parameters of Li_1.2_Ni_0.16_Mn_0.56_Co_0.08_O_2_

Parameter	Li_1.2_Ni_0.16_Mn_0.56_Co_0.08_O_2_	Reference^40^
*a* (Å)	4.9529(2)	4.944
*b* (Å)	8.5538(3)	8.561
*c* (Å)	5.0311(2)	5.025
*α* (°)	90	—
*β* (°)	109.246(4)	109.26
*γ* (°)	90	—
*V* (Å^3^)	201.237(16)	200.8

**Fig. 2:**
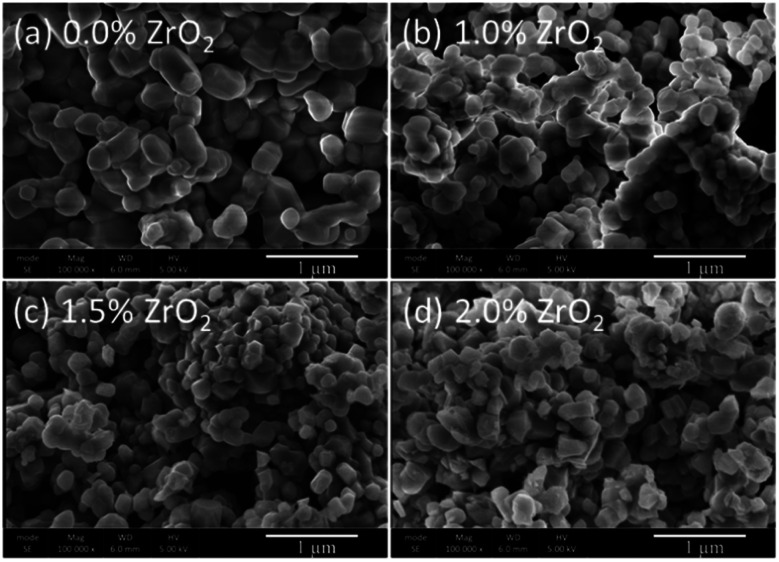
SEM images of (a) pristine and (b–d) ZrO_2_ coated Li_1.2_Ni_0.16_Mn_0.56_Co_0.08_O_2_ (ZrO_2_ = 1.0, 1.5 & 2.0 wt%).

The pristine and ZrO_2_ coated NMC (ZrO_2_ = 1.0, 1.5 & 2.0 wt%) materials have a rock-like spherical morphology (Fig. 2). It can be seen that the pristine NMC is composed of nanoparticles having a size in the range of ∼100–250 nm. On the other hand, the ZrO_2_ coated NMC materials show even smaller particles as compared to pristine NMC due to the ball milling effect and the presence of ZrO_2_ on the surface that may impeded the particle growth during the heat treatment process. This effect has already been discussed in details in the previous reports.^6^ It is pertinent to mention that the small particle size is considered helpful in improving the overall electrochemical performance, especially the rate capability of the material.^6^

**Fig. 3:**
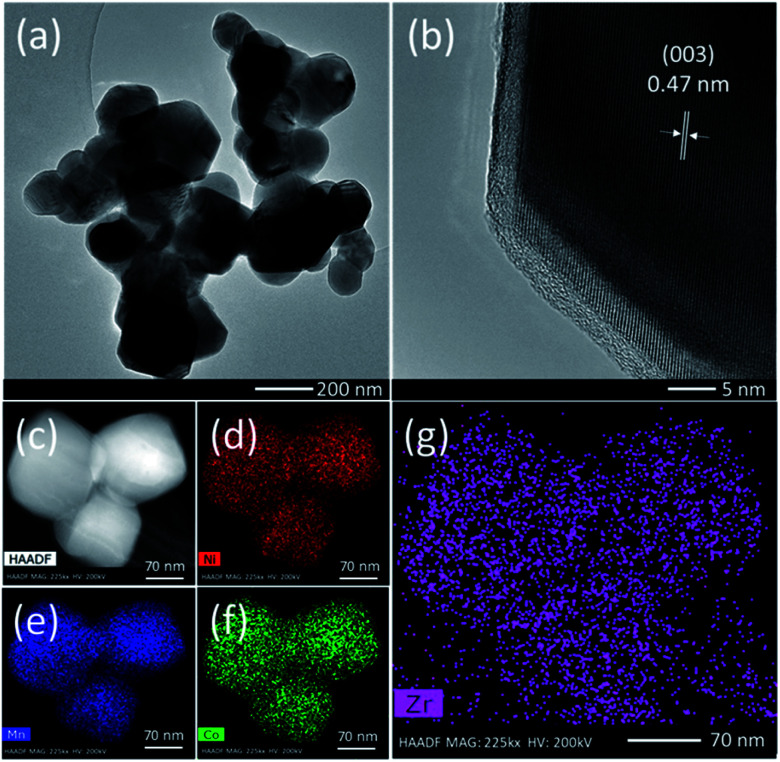
(a) TEM image, (b) HR-TEM image of particle (c–g) elemental mapping for different elements present in 1.5 wt% ZrO_2_ coated Li_1.2_Ni_0.16_Mn_0.56_Co_0.08_O_2_ (NMC).

TEM analysis was conducted in order to have more insight into the morphological features of the ZrO_2_ coated NMC material. The TEM images and elemental mapping of 1.5 wt% ZrO_2_ coated NMC is presented in Fig. 3. It can be clearly seen in Fig. 3(a) that the material is composed of nano-size particles which is consistent with the SEM images shown in Fig. 2. Furthermore, as discussed earlier, the particle size of ZrO_2_ coated materials is smaller than the pristine NMC as can be clearly seen through TEM images. The presence of ZrO_2_ coating on the surface of NMC particles impede the particle growth and thus ZrO_2_ coated materials have relatively smaller particle size. Fig. 3(b) shows an HR-TEM image of 1.5 wt% ZrO_2_ coated NMC where lattice fringes are clearly visible confirming the formation of highly crystalline materials. The lattice spacing is around 0.47 nm which correspond to (003) planes of the rhombohedral phase (*R*3̄*m*).^28,41^ Elemental mapping images in Fig. 3(d–g) show the distribution of Ni, Mn, Co, and Zr in 1.5 wt% ZrO_2_ coated NMC. All the elements are uniformly distributed throughout the particles, even the small amount of ZrO_2_ (1.5 wt%) shows homogeneous distribution on the particle surfaces rather than segregation at certain places. Thus, the coating strategy applied here successfully developed a homogenous ZrO_2_ coating layer on the particle surface. Fig. S-1† shows the TEM images and elemental mapping for 1.0 wt% ZrO_2_ coated NMC, which also indicates a uniform distribution of all elements on the particle surfaces.

**Fig. 4:**
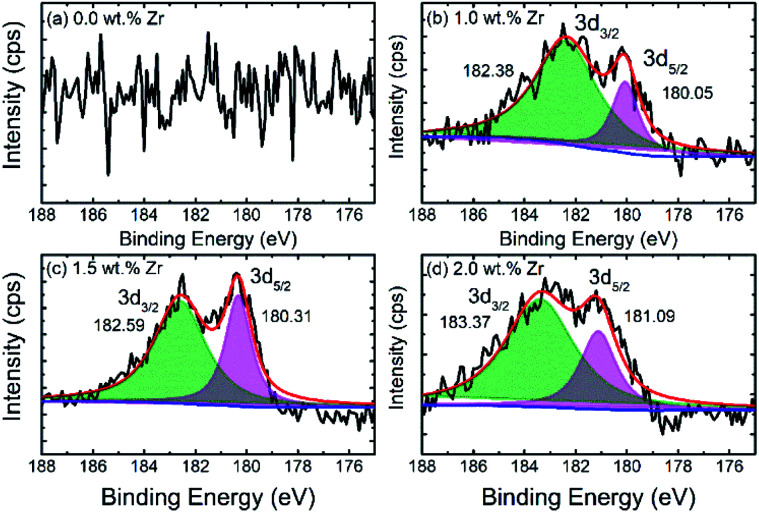
XPS spectra of (a) pristine and (b–d) ZrO_2_ coated Li_1.2_Ni_0.16_Mn_0.56_Co_0.08_O_2_ (ZrO_2_ = 1.0, 1.5 & 2.0 wt%) specifically showing spectra of zirconium.

The X-ray photoelectron spectroscopy (XPS) spectra of pristine and ZrO_2_ coated NMC materials (ZrO_2_ = 1.0, 1.5 & 2.0 wt%) are shown in Fig. 4. The high-resolution XPS spectra of zirconium (Zr) confirms the presence of ZrO_2_ on the particle surface as can be seen in Fig. 4(b–d). The binding energies for Zr are around 182.6 ± 0.5 eV and 180.3 ± 0.6 eV, respectively, that corresponds to Zr^4+^. The binding energies for ZrO_2_ coated NMC (ZrO_2_ = 1.0, 1.5 & 2.0 wt%) materials slightly vary from each other which may be due to the difference in the interactions between ZrO_2_ and Li-rich NMC materials. The XPS survey spectrums for pristine and ZrO_2_ coated materials are shown in Fig. S-2.† The binding energies of ZrO_2_ coated materials are compared in Table 2, with the reported values in the literature.^6^ This comparison indicates the close matching of the binding energy with the previously reported values. There is no Zr peak in the pristine NMC, as shown in Fig. 4(a) which confirms the absence of ZrO_2_ in this sample as this sample has not been coated with ZrO_2_.

**Table 2:** Binding energies of Zr in pristine and ZrO_2_ coated Li_1.2_Ni_0.16_Mn_0.56_Co_0.08_O_2_

Composition	Zr	
	3d_3/2_	3d_5/2_
Pristine NMC	—	—
1.0 wt% ZrO_2_-NMC	182.38	180.05
1.5 wt% ZrO_2_-NMC	182.59	180.31
2.0 wt% ZrO_2_-NMC	183.37	181.09
Reference^6^	183.11–183.53	180.80–181.95

**Fig. 5:**
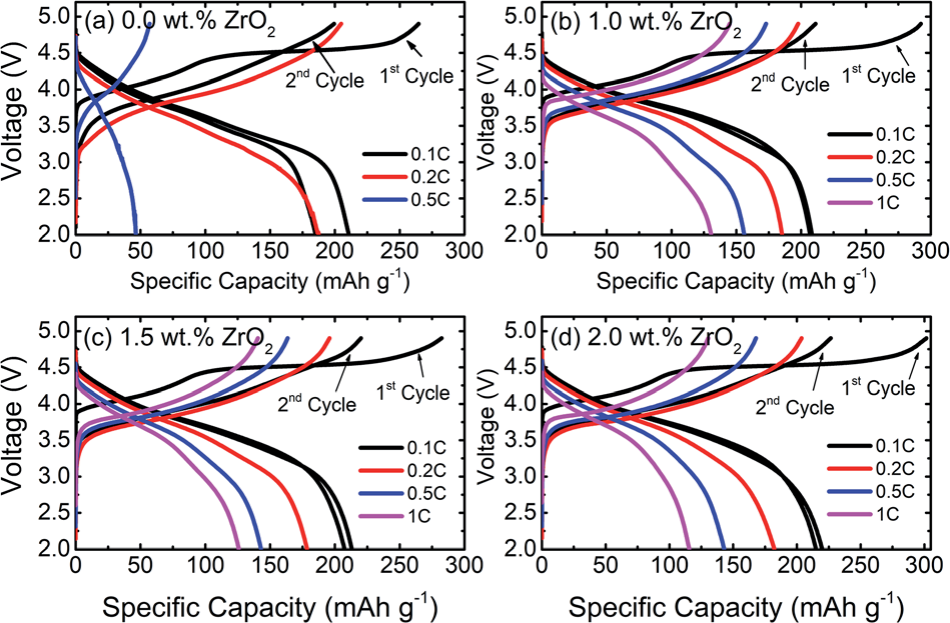
Galvanostatic charge/discharge curves of (a) pristine and (b–d) ZrO_2_ coated Li_1.2_Ni_0.16_Mn_0.56_Co_0.08_O_2_ (ZrO_2_ = 1.0, 1.5 & 2.0 wt%) at different C-rates.

The galvanostatic charge/discharge behaviour of pristine and ZrO_2_ coated NMC (ZrO_2_ = 1.0, 1.5 & 2.0 wt%) materials at different C-rates are presented in Fig. 5. It can be seen that there is a rapid capacity fading in pristine NMC with increasing C-rate attained a discharge capacity of 46 mA h g^−1^ at 0.5C, as shown in Fig. 5(a). On the other hand, ZrO_2_ coated NMC shows remarkably improved performance, as seen in Fig. 5(b–d). ZrO_2_ coated materials have higher discharge capacities and capacity retention at high C-rates compared to pristine NMC. The pristine material shows negligible capacity at 1C, whereas ZrO_2_ coated materials show a discharge capacity of over 110 mA h g^−1^ at 1C. The better performance of ZrO_2_ coated materials may be due to the prevention of direct contact of cathode material surface to the electrolyte, thus preventing unwanted side reactions and formation of unstable solid electrolyte interface (SEI) layer. It is well understood that the continuous growth of SEI consumes lithium and adversely affects battery performance.^26^

**Fig. 6:**
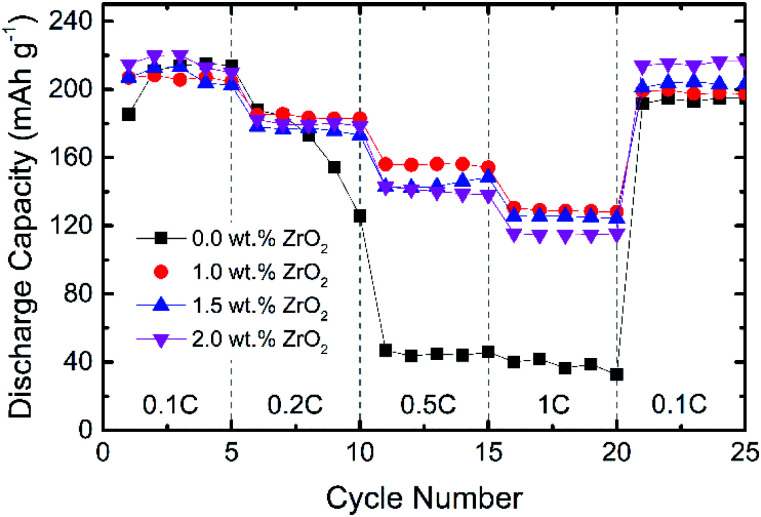
Rate capability of pristine and ZrO_2_ coated Li_1.2_Ni_0.16_Mn_0.56_Co_0.08_O_2_ (ZrO_2_ = 1.0, 1.5 & 2.0 wt%).

Fig. 6 shows the rate capability performance of pristine and ZrO_2_ coated NMC materials. All the materials show the initial discharge capacity between 205–218 mA h g^−1^ at a 0.1C rate. At higher C-rate, pristine NMC shows rapid capacity fading, whereas ZrO_2_ coated NMC shows better capacity retention. The discharge capacity for pristine NMC rapidly dropped to around 45 mA h g^−1^ at 0.5 and 1C. This may be due to the formation of an unstable solid electrolyte interface (SEI) layer which consumes active lithium and thus results in fast capacity degradation. The ZrO_2_ coated NMC (ZrO_2_ = 1.0, 1.5 & 2.0 wt%) shows improved rate capability, especially at a high C-rate with a discharge capacity of more than 110 mA h g^−1^ even at 1C. The 1.0 wt% ZrO_2_ coated NMC shows the best rate performance in terms of discharge capacity at 1C rate with a discharge capacity of 128 mA h g^−1^. The rate capability results (with increasing rates from 0.1C to 1C) demonstrate that the pristine NMC exhibits capacity retention of around 22% whereas 1.0 wt% ZrO_2_ coated NMC shows capacity retention of about 62%. The poor rate performance of pristine NMC may be either due to poor ionic diffusivity, slow phase boundary kinetics or increase charge transfer resistance at the electrode/electrolyte interface. This is further discussed in electrochemical impedance spectroscopy measurement section.

**Fig. 7:**
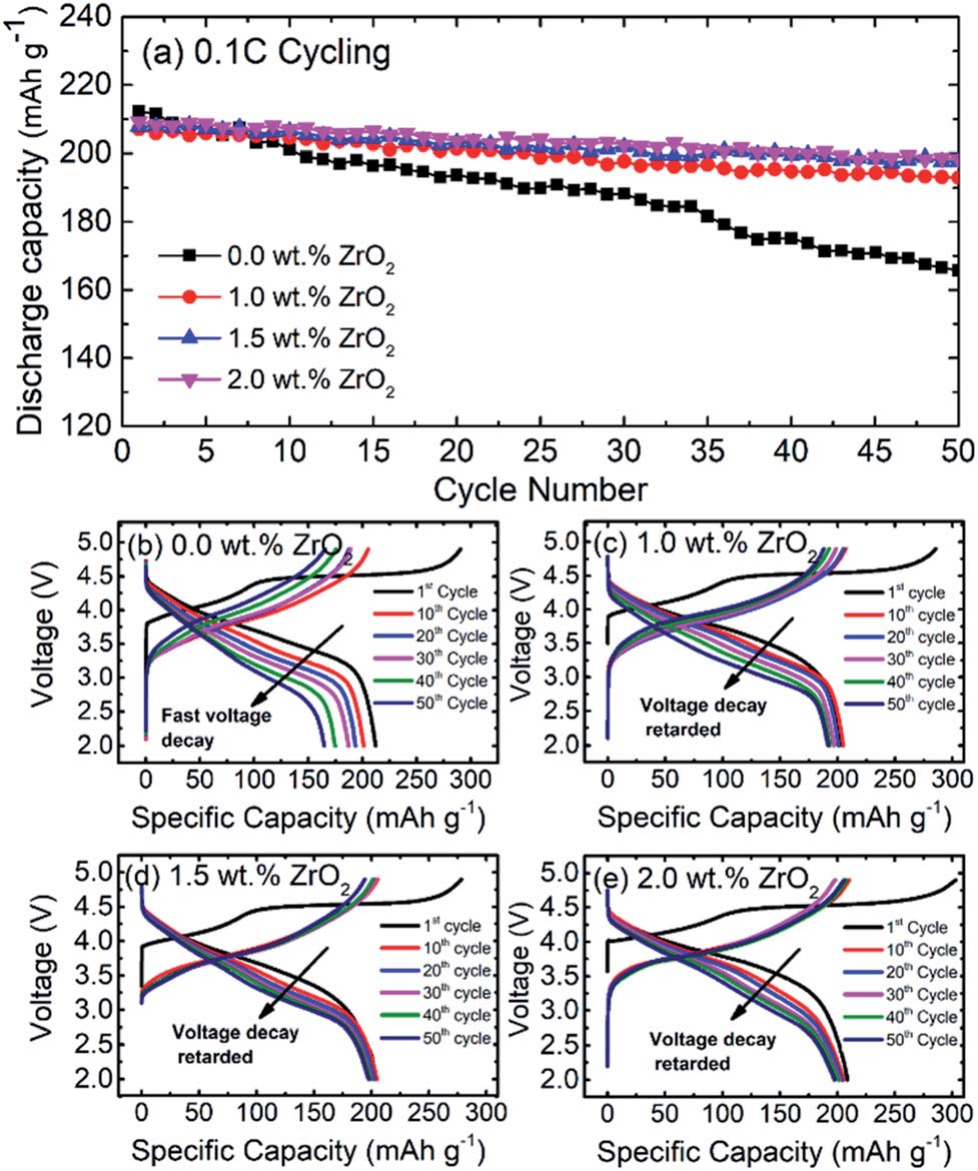
(a) Cycling behavior of pristine and ZrO_2_ coated Li_1.2_Ni_0.16_Mn_0.56_Co_0.08_O_2_ (ZrO_2_ = 1.0, 1.5 & 2.0 wt%) at 0.1C for 50 cycles, (b–e) galvanostatic charge/discharge curves at 1^st^, 10^th^, 20^th^, 30^th^, 40^th^ and 50^th^ cycles respectively.

Fig. 7(a) shows the cycling performance of pristine and ZrO_2_ coated NMC (ZrO_2_ = 1.0, 1.5 & 2.0 wt%). The cells were charged and discharged at a 0.1C rate for 50 cycles. It is observed that the capacity of pristine NMC rapidly decreases with an increasing number of cycles whereas ZrO_2_ coated NMC (ZrO_2_ = 1.0, 1.5 & 2.0 wt%) shows slow capacity fade over 50 cycles. The reason for the fast capacity fading in pristine NMC may be due to either structural instability or development of unstable solid electrolyte interface (SEI). On the other hand, ZrO_2_ coating seems to protect the surface of NMC, thus improving the cycling performance. However, 1.5 and 2.0 wt% ZrO_2_ coated NMC exhibited the best capacity retention of around 95%. On the other hand, 1.0 wt% ZrO_2_ coated NMC exhibited capacity retention of around 92.7% after 50 cycles.

Fig. 7(b–e) Shows the galvanostatic charge/discharge profiles of pristine NMC and ZrO_2_ coated NMC materials after 1^st^, 10^th^, 20^th^, 30^th^, 40^th^ and 50^th^ cycle. The cells were charge between 2.0–4.9 V. It can be seen in Fig. 7(b) that there was fast capacity fade and voltage decay for the pristine NMC. On the other hand, ZrO_2_ coated NMC (ZrO_2_ = 1.0, 1.5 & 2.0 wt%) show improved performance with respect to capacity retention and voltage decay with successive cycling (Fig. 7(c–e)). The voltage decay is retarded with ZrO_2_ coating, and slow voltage decay is observed. The 1.5 wt% ZrO_2_ coated NMC material showed the best voltage retention as compared to 1.0 and 2.0 wt% ZrO_2_ coated materials. Therefore, ZrO_2_ coating has enhanced the structural stability of the material and helped to improve the electrochemical performance of the material.

The GITT measurements were performed to extract different resistive processes as a function of the state of charge (SOC). The EIS spectra were recorded in the half-cell configuration at different states of charges and discharges. The impedance spectrum at 40% state of charge is shown in the supplementary Fig. S-3.† The cell was held at the open-circuit voltage (OCV) for three hours with a voltage decay rate of 2 mV h^−1^ at the end of the rest interval. The measured Nyquist plots of coated Li_1.2_Ni_0.16_Mn_0.56_Co_0.08_O_2_ consist of the following features:

(1) a high-frequency intercept represents the ionic resistance of the electrolyte along with a small contribution of the SEI layer;

(2) the first semi-circle at the medium–high frequencies, which represents the electronic conductivity of the material along with the charge transfer resistance at the lithium/electrolyte interface;

(3) a second semi-circle at the medium-low frequencies, which represents the charge transfer reaction at the Li_1.2_Ni_0.16_Mn_0.56_Co_0.08_O_2_/electrolyte interface and,

(4) a Warburg response is presented at the lower frequency region of the plots. Similar impedance spectra were measured at other states of charge/discharge process.

**Fig. 8:**
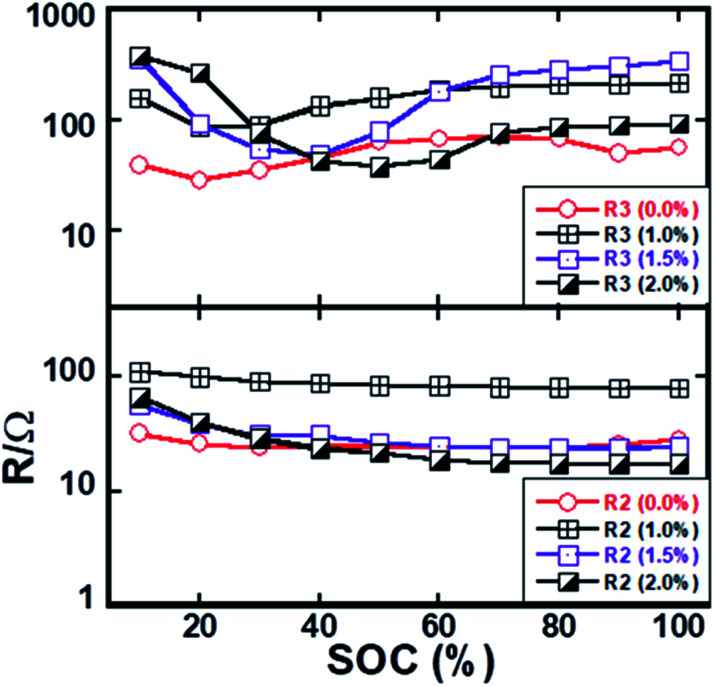
(a) Interfacial charge transfer resistances of pristine and ZrO_2_ coated NMC electrodes. Data extracted from the half-cell configuration by EIS measurements as a function of the state of charge.

Different resistances were extracted by fitting the spectra using the equivalent circuit as shown in Fig. S-4,† and the obtained resistance data are displayed in Fig. 8 as a function of the state of charge (SOC) excluded with the ohmic resistance of electrolyte solution. The equivalent circuit model comprises eight circuit elements which was used to fit the EIS data. L1 represents the induction of the cell components and wire. The resistances of first semi-circles (*R*_2_) slightly decrease with the degree of delithiation up to around 20% state of charge and after that *R*_2_ values are almost constant with further delithiation. On the other hand, the resistance of the second semi-circle (*R*_3_) initially decreases with charging to a minimum value and after that, gradually increases and almost kept constant value on further delithiation. Three coated materials exhibit a similar trend of *R*_3_ value as a function of SOC. It should be noted that the resistance (*R*_3_) of lower frequency semi-circle is significantly higher than the resistance (*R*_2_) that is associated with the higher frequency semi-circle. The *R*_2_ should not be changed with the state of charge considering that it is only associated with the charge transfer reaction at the lithium/electrolyte interface. However, the electronic conductivity of active material changes with the state of charge, which has an impact on both semi-circles. The change of *R*_2_ as a function of SOC is associated electronic conductivity of active particles since the capacitance value of change transfer at lithium/electrolyte interface and electronic conductivity of active particles almost the same. It has an impact on the charge transfer resistance at *R*_3_/electrolyte interface since the electronic conductivity of active particle influences charge transfer kinetics.

The slight decrease of *R*_2_ with SOC should be due to the electronic conductivity of the active particles as the result of the mixed-valence state that is formed during delithiation. Charge transfer kinetics at electrode–electrolyte interfacial can be improved with the increase of electronic conductivity of active materials as reported in our previous report.^42^ It can also be seen in Fig. 8 that the resistance *R*_3_ is higher for the higher lithium concentrations interval. Indeed, the electronic conductivity of the material is relatively low in the fully lithiated phase.^43^ The EIS results likely imply that the interfacial charge transfer resistance is rate-limiting particularly, at the cathode/electrolyte interface. The coated and pristine materials exhibit similar magnitude of *R*_2_ and *R*_3_ values indicating similar interfacial charge transfer kinetics. The rate capability should not be much different. Nonetheless, the coated electrode exhibits better electrochemical performances than the pristine electrode. Such observation indicates the interface degradation rather than interfacial kinetic.

**Fig. 9:**
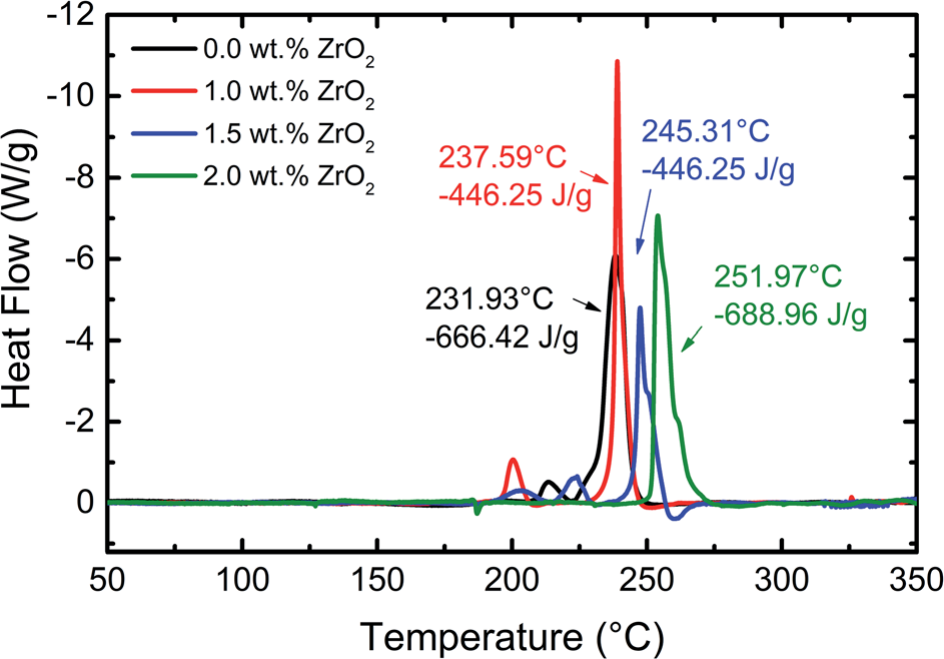
DSC profiles of pristine and ZrO_2_ coated Li_1.2_Ni_0.16_Mn_0.56_Co_0.08_O_2_ electrodes with a scan rate of 5 °C min^−1^. The cells were charged to 4.7 V at 0.1C.

The thermal stability of pristine and ZrO_2_ coated NMC electrodes was investigated using DSC measurements after being charged to 4.7 V, and the obtained results are shown in Fig. 9. All the materials exhibit strong exothermic peaks beyond 231 °C. However, the weak exothermic peaks have appeared between 200 °C and 225 °C which may be due to, (1) decomposition of PVDF binder, (2) phase transformation of active materials or (3) some changes in the absorbed electrolyte. It can be seen from Fig. 9 that the thermal stability of ZrO_2_ coated NMC electrodes increased compared with the pristine electrode. The difference in onset temperature between the pristine and 2 wt% ZrO_2_ coated electrode is around 20 °C which is an indication of improvement of thermal stability of ZrO_2_ coated NMC electrode. The onset temperature increased gradually with increasing the amount of coating material. However, the total heat flow exhibits anomalous behaviour. This might be due to different amounts of samples loaded in the DSC crucible and different extent of reaction rate. Table 3 shows the onset temperature and heat generation for all the materials. These results clearly demonstrated the improved thermal stability of ZrO_2_ coated NMC electrodes.

**Table 3:** Onset temperature and heat generation for pristine and ZrO_2_ coated NMC materials

Sample	Onset temperature (°C)	Heat generation (J g^−1^)
0.0 wt% ZrO_2_	231.93	666.42
1.0 wt% ZrO_2_	237.59	487.31
1.5 wt% ZrO_2_	245.31	446.25
2.0 wt% ZrO_2_	251.97	688.96

## Conclusions

The ZrO_2_ coated Li_1.2_Ni_0.16_Mn_0.56_Co_0.08_O_2_ (where ZrO_2_ = 1.0, 1.5 & 2.0 wt%) materials were synthesized using sol–gel assisted ball mill process. The XRD and Rietveld refinement analysis confirmed the presence of phase pure materials. SEM and TEM images confirmed the rock-like particle morphology with an average particle size between 100–250 nm. TEM elemental mapping images confirmed the homogeneous distribution of Zr on the surface of the particles. The ZrO_2_ coated NMC materials exhibit better electrochemical performance than the pristine NMC material. The observed poor rate capability, fast capacity and voltage fade for the pristine material are due to unwanted side reaction with electrolyte solution and structural instability during the cycling process. The ZrO_2_ coating helps to suppress these unwanted side reactions and thus result in improved performance. Finally, ZrO_2_ coated NMC materials demonstrate improved thermal stability with an increase in onset temperatures and decrease in heat generation for ZrO_2_ coated NMC materials.

## Conflicts of interest

There are no conflicts to declare.

## Supplementary Material

RA-010-D0RA02060E-s001

## References

[cit1] Armand M., Tarascon J.-M. (2008). Nature.

[cit2] Whittingham M. S. (2008). MRS Bull..

[cit3] Whittingham S. (2008). MRS Bull..

[cit4] Nitta N., Wu F., Lee J. T., Yushin G. (2015). Mater. Today.

[cit5] Larcher D., Tarascon J.-M. (2015). Nat. Chem..

[cit6] Nisar U., Amin R., Essehli R., Shakoor R. A., Kahraman R., Kim D. K., Khaleel M. A., Belharouak I. (2018). J. Power Sources.

[cit7] Nisar U., Amin R., Shakoor A., Essehli R., Al-Qaradawi S., Kahraman R., Belharouak I. (2018). Emergent Mater..

[cit8] Nisar U., Gulied M. H., Ahmad Z., Shakoor R. A., Al-Qaradawi S., Soliman A., Essehli R., Alashraf A., Kahraman R. (2018). RSC Adv..

[cit9] Palacín M. R. (2009). Chem. Soc. Rev..

[cit10] Kraytsberg A., Ein-Eli Y., Kraytsberg A., Ein-Eli Y. (2012). Adv. Energy Mater..

[cit11] Xu B., Qian D., Wang Z., Meng Y. S. (2012). Mater. Sci. Eng., R.

[cit12] Nisar U., Al-Hail S. A. J. A., Petla R. K., Shakoor R. A., Essehli R., Kahraman R., AlQaradawi S. Y., Kim D. K., Belharouak I., Amin M. R. (2019). ACS Appl. Energy Mater..

[cit13] Tarascon J.-M. (2010). Philos. Trans. R. Soc., A.

[cit14] Julien C. M. and Mauger A., Review of 5-V electrodes for Li-ion batteries: Status and trends, 2013, vol. 19

[cit15] He P., Yu H., Li D., Zhou H. (2012). J. Mater. Chem..

[cit16] Liu X., He P., Li H., Ishida M., Zhou H. (2012). J. Alloys Compd..

[cit17] Yuan L. X., Wang Z. H., Zhang W. X., Hu X. L., Chen J. T., Huang Y. H., Goodenough J. B. (2011). Energy Environ. Sci..

[cit18] Lee M., Lee S., Oh P., Kim Y., Cho J. (2013). Nano Lett..

[cit19] Da Li Y., Zhao S. X., Nan C. W., Li B. H. (2011). J. Alloys Compd..

[cit20] Thackeray M. M., Kang S.-H., Johnson C. S., Vaughey J. T., Benedek R., Hackney S. a. (2007). J. Mater. Chem..

[cit21] Rozier P., Tarascon J. M. (2015). J. Electrochem. Soc..

[cit22] Pimenta V., Sathiya M., Batuk D., Abakumov A. M., Giaume D., Cassaignon S., Larcher D., Tarascon J., De Picardie U., Verne J. (2017). Chem. Mater..

[cit23] Shang H., Ning F., Li B., Zuo Y., Lu S., Xia D. (2018). ACS Appl. Mater. Interfaces.

[cit24] Hu S. , Pillai A. S., Liang G., Pang W. K., Wang H., Li Q. and Guo Z., Li-Rich Layered Oxides and Their Practical Challenges: Recent Progress and Perspectives, Springer Singapore, 2019

[cit25] Andersson a. M., Abraham D. P., Haasch R., MacLaren S., Liu J., Amine K. (2002). J. Electrochem. Soc..

[cit26] Yu X., Manthiram A. (2018). Energy Environ. Sci..

[cit27] Minato T., Abe T. (2017). Prog. Surf. Sci..

[cit28] Xie Q., Zhao C., Hu Z., Huang Q., Chen C., Liu K. (2015). RSC Adv..

[cit29] Gao Y., Xu P., Chen F., Ding C., Chen L., Li D. (2016). RSC Adv..

[cit30] He X., Wang J., Kloepsch R., Krueger S., Jia H., Liu H., Vortmann B., Li J. (2014). Nano Res..

[cit31] Wu F., Li N., Su Y., Shou H., Bao L., Yang W., Zhang L., An R., Chen S. (2013). Adv. Mater..

[cit32] Jiang K. C., Wu X. L., Yin Y. X., Lee J. S., Kim J., Guo Y. G. (2012). ACS Appl. Mater. Interfaces.

[cit33] Wu F., Li N., Su Y., Zhang L., Bao L., Wang J., Chen L., Zheng Y., Dai L., Peng J., Chen S. (2014). Nano Lett..

[cit34] Myung S.-T., Amine K., Sun Y.-K. (2010). J. Mater. Chem..

[cit35] Mauger A., Julien C. (2014). Ionics.

[cit36] Li X., Zhang K., Mitlin D., Yang Z., Wang M., Tang Y., Jiang F., Du Y., Zheng J. (2018). Chem. Mater..

[cit37] Kobayashi G., Irii Y., Matsumoto F., Ito A., Ohsawa Y., Yamamoto S., Cui Y., Son J. Y., Sato Y. (2016). J. Power Sources.

[cit38] Liao J. Y., Manthiram A. (2015). J. Power Sources.

[cit39] Wang Z., Liu E., Guo L., Shi C., He C., Li J., Zhao N. (2013). Surf. Coat. Technol..

[cit40] Nayak P. K., Grinblat J., Levi E., Levi M., Markovsky B., Aurbach D. (2017). Phys. Chem. Chem. Phys..

[cit41] Shi S., Wang T., Cao M., Wang J., Zhao M., Yang G. (2016). ACS Appl. Mater. Interfaces.

[cit42] Ben Yahia H., Essehli R., Amin R., Boulahya K., Okumura T., Belharouak I. (2018). J. Power Sources.

[cit43] Amin R., Chiang Y. M. (2016). J. Electrochem. Soc..

