# Supplementary material for: Impact of surface coating on electrochemical and thermal behaviors of a Li-rich Li1.2Ni0.16Mn0.56Co0.08O2 cathode
Source: RSC Adv. 2020 Apr 17;10(26):15274–81. doi: 10.1039/d0ra02060e (PMC9052460; doi:10.1039/d0ra02060e)
Supplement: RA-010-D0RA02060E-s001 [file RA-010-D0RA02060E-s001.pdf]

Electronic Supplementary Material (ESI) for RSC Advances.
This journal is © The Royal Society of Chemistry 2020

Supplementary Information

Impact of Surface Coating on Electrochemical and Thermal Behaviors of Li-rich $Li_{1.2}Ni_{0.16}Mn_{0.56}Co_{0.08}O_2$ Cathode

Umair Nisar,[a] Ramesh Petla,[b] Sara Ahmad Jassim Al-Hail,[b] Aisha Abdul Quddus,[c] Haya Monawwar,[d] Abdul Shakoor,[a, *] Rachid Essehli,[e] Ruhul Amin,[e, *]

[a]*Center for Advanced Materials (CAM), Qatar University, Doha, Qatar*

[b]*Qatar Environment and Energy Research Institute (QEERI), Hamad Bin Khalifa University, Qatar Foundation, 34110, Doha, Qatar*

[c]*Department of Chemical Engineering, College of Engineering, Qatar University, Doha, Qatar*

[d]*Department of Electrical Engineering, College of Engineering, Qatar University, Doha, Qatar*

[e]*Energy and Transportation Science Division, Oak Ridge National Laboratory, Oak Ridge, TN, USA*

Corresponding authors:

aminr@ornl.gov (Dr. Ruhul Amin)

shakoor@qu.edu.qa (Dr. R. A. Shakoor)

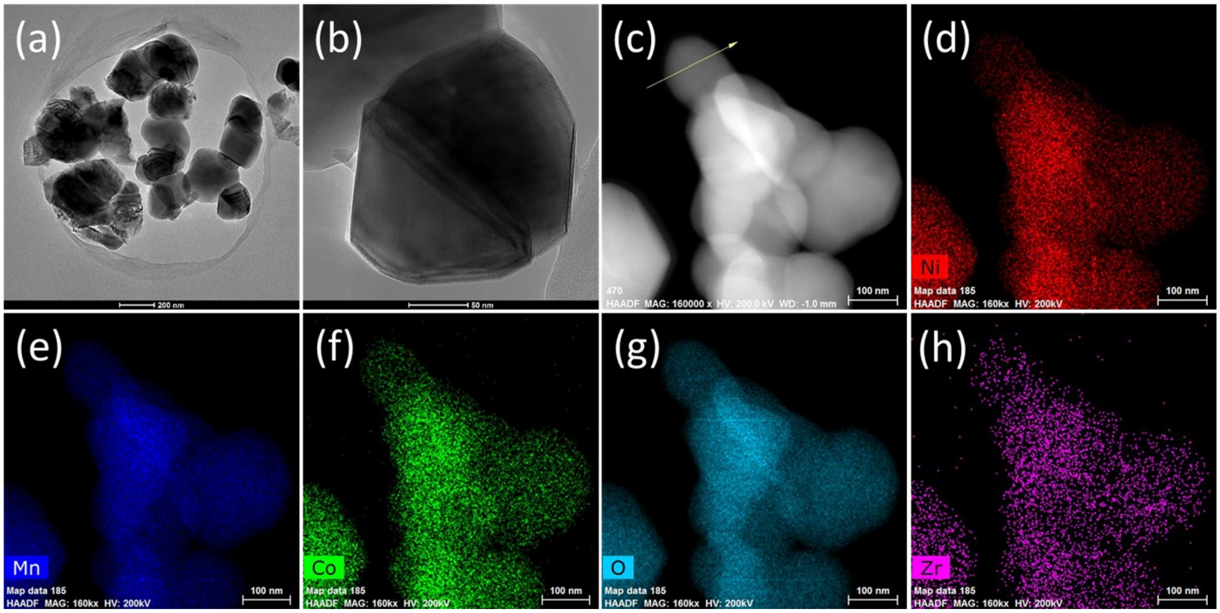

Fig. S-1. TEM and elemental images of 1wt.% ZrO_2 coated $Li_{1.2}Ni_{0.16}Mn_{0.56}Co_{0.08}O_2$ (NMC).

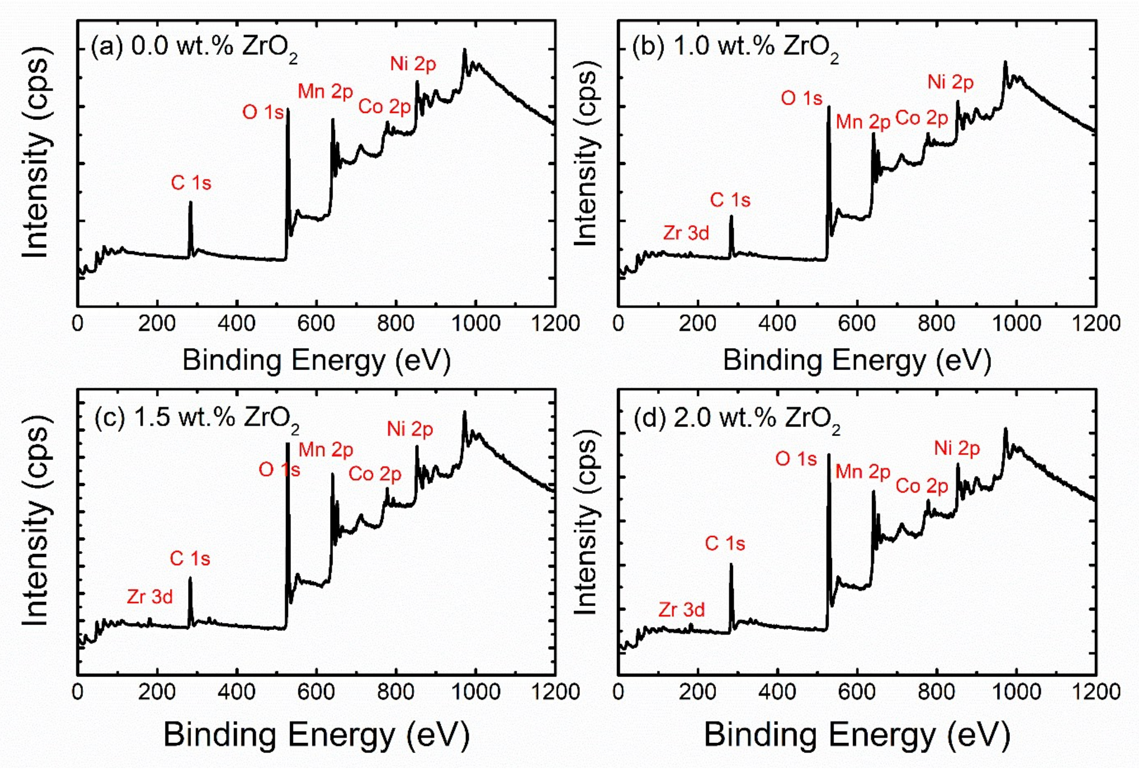

Fig. S-2. XPS survey patterns for (a) pristine and (b-d) ZrO_2 coated $Li_{1.2}Ni_{0.16}Mn_{0.56}Co_{0.08}O_2$ (ZrO_2=1.0, 1.5 & 2.0 wt.%).

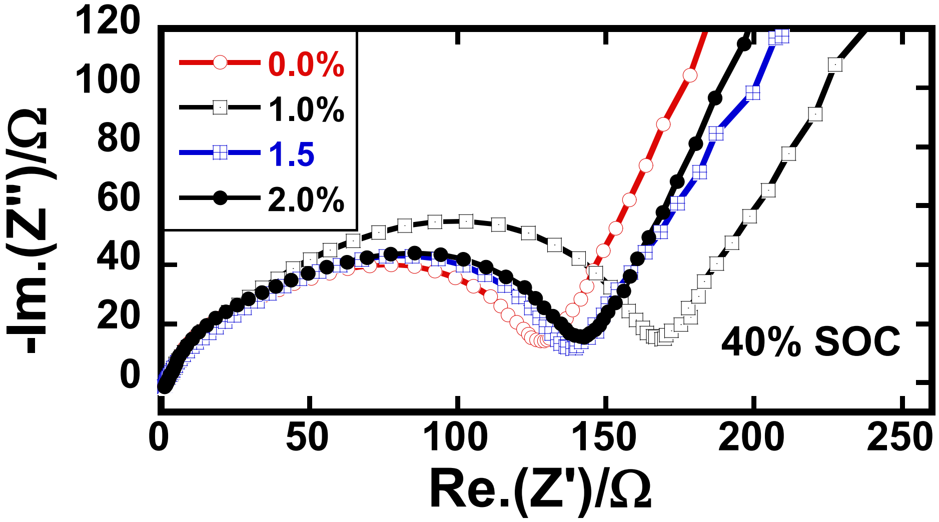

Fig. S-3 Impedance spectra of ZrO_2 coated $Li_{1.2}Ni_{0.16}Mn_{0.56}Co_{0.08}O_2$ (ZrO_2=0.0%, 1.0%, 1.5% & 2.0 wt.%) at 40% state of charge.

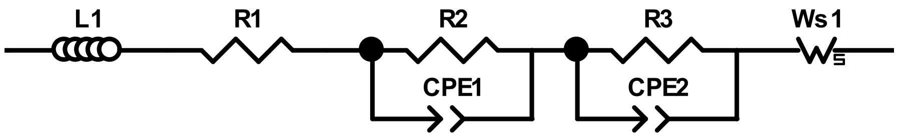

Fig. S-4 Equivalent circuit used to evaluate the differences resistances as a function of state of charged and discharged.
